# Current knowledge and practice of post-stroke unilateral spatial neglect rehabilitation: A cross-sectional survey of South African neurorehabilitation physiotherapists

**DOI:** 10.4102/sajp.v78i1.1624

**Published:** 2022-03-30

**Authors:** Chuka I. Umeonwuka, Ronel Roos, Veronica Ntsiea

**Affiliations:** 1 Department of Physiotherapy, Faculty of Health Sciences, University of the Witwatersrand, Johannesburg, South Africa

**Keywords:** unilateral spatial neglect, South Africa, knowledge, stroke, physiotherapists

## Abstract

**Background:**

Unilateral spatial neglect (USN) affects the rehabilitation process leading to poor functional outcomes after stroke. South African physiotherapists’ level of uptake of available evidence in USN rehabilitation and the barriers they encounter are not known.

**Objectives:**

To evaluate knowledge, current practice enablers and barriers to USN management in stroke survivors amongst physiotherapists in South Africa.

**Methods:**

Our cross-sectional survey used a total sampling technique. Questionnaires were sent to neurorehabilitation physiotherapists in South Africa. Descriptive and inferential statistics analysed the data.

**Results:**

The overall knowledge score of USN was 14.11 ± 5.23 of a total of 25. The knowledge was good for definitions of USN; moderate for incidences, causes, screening, diagnosis and prognosis of USN and poor for pharmacological approaches to the management of USN. A significant low positive correlation between respondents’ age (*r* = 0.46; *p* = 0.016) and years of practice as a physiotherapist (*r* = 0.43; *p* = 0.026) and knowledge of USN was found. The most frequently utilised intervention was constraint-induced movement therapy; the commonly utilised assessment tool was the comb and razor test. ‘Inadequate therapy time’ (55.56%) and ‘lack of relevant equipment for rehabilitation of USN’ (38.89%) were identified as major barriers to USN rehabilitation. Major enablers to USN rehabilitation were the ‘presence of multidisciplinary stroke team in clinical practice’ (83.35%) and ‘availability of adequate staff’ (76.47%).

**Conclusion:**

Physiotherapists demonstrated a fair knowledge of USN although knowledge about pharmacological management of USN was modest. Current practice in post-stroke USN by South African neuro-physiotherapists follows current evidence and practice guidelines.

**Clinical implication:**

Our study shows the level of knowledge and current practice of post-stroke USN rehabilitation. The demonstrated fair knowledge of USN may be improved through training, curriculum modifications or continuing professional development. Identified barriers to the rehabilitation of post-stroke USN can assist health policy, managers and clinicians to improve stroke-specific care.

## Introduction

Unilateral spatial neglect (USN) is:

[*A*] failure to report, respond or orient to stimuli presented in the side of space contralateral to the injured cerebral hemisphere, which cannot be explained by primary sensory or motor deficit. (Heilman, Valenstein & Watson 2000, p. 2)

Spatial neglect occurs after a stroke or degenerative disease and is a multifaceted and disabling condition (Andrade et al. 2010).

Incidence of neglect differs extensively in reported studies, and this variability is between 12% and 95% (Robertson & Halligan 1999). This variability of USN incidence is because of a plethora of factors such as the operational definition of USN, assessment type to identify USN and heterogeneity of USN presentation (Ting et al. 2011). In many patients post-stroke, the severity of neglect symptoms persist chronically (Farnè et al. 2004; Katz et al. 1999) with a negative affectation in almost every activity that contributes to daily living and rehabilitation outcomes such as postural balance (Gottlieb & Levine 1992; Pérennou 2006; Taylor, Ashburn & Ward 1994; Van Nes et al. 2009), functional mobility (Berti et al. 2002; Tromp, Dinkla & Mulder 1995), quality of life (Franceschini et al. 2010; Sobrinho et al. 2018), ADLs (Cherney et al. 2001; Jehkonen et al. 2000, 2001; Vossel et al. 2013) and hospital stay duration (Chen et al. 2015; Hammerbeck et al. 2019; Wee & Hopman 2008).

Several interventions to ameliorate USN have been proposed by researchers (Luauté et al. 2006; Umeonwuka, Roos & Ntsiea 2020). Strategies for USN symptom amelioration are broadly classified into bottom-up or top-down methods (Marshall 2009). Treatment options have metamorphosed in the last decades from a more conventional and conservative approach to the use of technology combining more than one treatment approach (Umeonwuka et al. 2020), and they include prism adaptation, combination therapy, constraint-induced movement therapy (CIMT), transcutaneous electrical nerve stimulation (TENS), transcranial magnetic stimulation, virtual reality, visual scanning exercise and trunk rotation exercise.

Current stroke treatment guidelines recommend the all-inclusive and timeous screening and diagnosis of USN as a critical part of post-stroke care (Bryer et al. 2010; National Stroke Foundation 2010; Royal College of Physicians 2012; Winstein et al. 2016). However, strict adherence to these protocols is not guaranteed, and translational gaps exist between theory and practice in the management of post-stroke USN (Checketts et al. 2020). Identified assessment currently utilised by rehabilitation experts in clinical practice includes cognitive, functional and neuro-imaging (Checketts et al. 2020).

In South Africa, a study by Ntsiea (2019), that collated South African physiotherapy stroke rehabilitation services and research, identified saccadic eye movement priming with visual scanning exercise as an effective physiotherapy intervention for USN. However, no study has examined the uptake of interventions identified as effective in the treatment of USN by South African neurorehabilitation physiotherapists.

An understanding of the therapists’ knowledge, practice and barriers they encounter in the treatment of USN will enable the neurorehabilitation team including the physiotherapy educators to better strategise and plan to achieve better outcomes in USN-specific treatment post-stroke. With the foregoing in mind, our study was conceptualised to evaluate physiotherapists’ knowledge, current practice and barriers and facilitators to the rehabilitation of USN in South Africa.

## Methods

A cross-sectional national survey of the members of the neurology speciality interest group of the South African Society of Physiotherapists (SASP) was conducted from November 2018 to June 2019. Community service physiotherapists were not included. Community service physiotherapists were excluded because of their modest work experience in neurorehabilitation, given that they rotate through different departments and hospital wards during their community service year. A total sampling technique was used as all 277 members of the South African Society of Physiotherapy neurology special interest group were invited to participate in our study.

### Data collection tool

This was a questionnaire on knowledge, current practice, barriers and enablers to USN. The questionnaire was validated by three research experts with doctorate degrees (two neuro physiotherapists and a public health expert) who were knowledgeable in questionnaire design and experts in stroke rehabilitation. It was also pilot tested on eight consenting clinicians in stroke rehabilitation. The questionnaire took approximately 15–20 min to complete and consisted of four sections (Section A to D). Section A elicited questions on the respondent’s demographic characteristics. Section B contained 25 items for assessing the knowledge on USN developed for our study using a previous study by Petzold as a guide in developing the questionnaire (Petzold et al. 2012). The questionnaire included questions on (1) USN problem identification, (2) USN assessment use, (3) USN intervention use and (4) knowledge of USN and best practice recommendations including the anatomy of neglect. Each item has three responses (‘Agree’, ‘Disagree’ or ‘Undecided’). The maximum score is 25, and the minimum score is 0. An ‘Agree’ response to a correct statement is scored 1, a ‘Disagree’ response to a wrong statement is also scored 1 and a ‘Disagree’ response to a correct statement is scored 0. An ‘Agree’ response to a wrong statement is scored 0 and an ‘Undecided’ response is disregarded. A knowledge score of < 10 indicates poor knowledge; 10–19 indicates moderate knowledge, whilst 20–25 represents good knowledge. Item-by-item responses as well as the summed scores for knowledge of physiotherapists on USN are presented. The summed score was for inferential statistic comparison with physiotherapist’s knowledge of USN, whilst item-by-item responses were to show group responses to the specific knowledge of USN questions.

Section C assessed the physiotherapist’s current practice of USN. The questions were structured to decipher the current assessment strategy, treatment approach, referral or teamwork in the treatment of post-stroke USN. Finally, section D contained multiple-choice questions on enablers and barriers already identified in the literature on stroke rehabilitation (Ogourtsova, Archambault & Lamontagne 2019; Petzold et al. 2014). The questions on barriers consist of therapist, institutional, client suitability and equipment factors.

Similarly, therapists were asked to rate barriers or enablers to the treatments of post-stroke USN on a scale of 1–5 with 1 being the greatest barrier or enabler and 5 being the least barrier or enabler. The scoring was done so that 1–2 = major barrier or facilitator, 3 = moderate barrier or facilitator and 4–5 = minor barrier or facilitator.

The interclass correlation of this questionnaire (α) yielded 0.93 (excellent reliability), 0.72 (moderate reliability) and 0.64 (moderate reliability) for sections B, C and D, respectively. An inter-class correlation coefficient (ICC) value less than 0.5 indicates poor reliability, a value between 0.5 and 0.75 indicates moderate reliability, a value between 0.75 and 0.9 indicates good reliability and a value greater than 0.90 indicates excellent reliability (Koo & Li 2016)

### Data collection procedure

Study data were collected and managed using Redcap (Research Electronic Data Capture), an electronic data capture tool hosted at the University of the Witwatersrand (Harris et al. 2009). A survey link containing our study questionnaire was sent through the SASP to the 277 members of the SASP neurology speciality interest group.

### Statistical analysis

A version 25.0 of IBM SPSS Statistics for Macintosh was used for the analysis of the data (IBM 2017). Means and standard deviations were used to summarise continuous data, whilst frequencies and percentages were used to summarise the demographic characteristics of therapists. Responses to questions on the current practice, barriers and enablers of post-stroke USN were summarised using descriptive statistics of frequencies and percentages.

To explore relationships between demographic characteristics of the main outcome measure (knowledge of USN), inferential statistics were used. Spearman rank correlation was used to measure the degree of association of continuous variables that were not normally distributed, whilst the student t-test was used to compare the means of continuous normally distributed data. The ANOVA test was used to check whether the means of two or more groups were significantly different. A significance level of 0.05 was used. Item-by-item responses of the knowledge section of the questionnaire as well as the summed scores for knowledge of USN are presented to allow readers to examine the data in both ways.

### Ethical considerations

The Human Research Ethics Committee of the University of the Witwatersrand provided ethical clearance for our study. Informed consent was obtained electronically from the physiotherapists by agreeing to continue with the online survey. As a process of informed consent, all physiotherapists were informed of our study’s purpose and that their participation was voluntary and anonymous.

## Results

### Respondents’ characteristics

A total of 277 physiotherapists who are members of the neurology interest group of the SASP were invited to participate. Fifty physiotherapists responded to the questionnaire, and 28 questionnaires were complete and valid. Female respondents were in the majority (*n* = 25; 89.29%), whilst the median age was 34.5 (22–61) years. Most respondents worked in urban settings. (*n* = 23; 82.14%). Most had a bachelors’ degree as the highest educational level (*n* = 14; 50%), whereas 7 respondents (25%) possessed a master’s degree and six (21.43%) possessed a doctorate (PhD). However, only 9 (32.14%) of the therapists had a post-graduate degree certification in neurological rehabilitation. Most of the respondents worked full time in neurorehabilitation (*n* = 16; 76.19%). The median years of practice was 12 (0–40) years, whilst the median duration of practice in neurorehabilitation of the respondents was 10.5 (1–30) years. Table 1 outlines the characteristics of the respondents.

**TABLE 1:** Socio-demographic characteristics of participants (*n* = 28).

Variable	Value			
	Median	Range	*n*	%
**Age**				
Median (range) in years	34.5	22–61	-	-
**Gender**				
Male	-	-	3	10.71
Female	-	-	25	89.29
**Setting**				
Semi-urban	-	-	5	17.86
Urban	-	-	23	82.14
Years of practice as a physiotherapist Median (range) in years	12	0–40	-	-
**Educational level**				
Bachelors	-	-	14	50.00
Post-graduate diploma	-	-	1	3.57
Master’s Degree	-	-	7	25.00
Doctorate	-	-	6	21.43
**Clinical practice setting**				
Hospital	-	-	9	32.14
Rehabilitation centre	-	-	13	46.43
Out-patient Department	-	-	4	14.29
Domiciliary/Home health	-	-	2	7.14
**Postgraduate certification in neurological rehabilitation**				
Yes	-	-	9	32.14
No	-	-	19	67.86
**Work full time in neurorehabilitation**				
Yes	-	-	16	76.19
No	-	-	5	23.81
Duration of practice in neurorehabilitation unit, median (range) in years	10.5	1–30	-	-
**Level**				
Physiotherapist	-	-	19	67.86
Chief Physiotherapist	-	-	5	17.86
Others (Academics who are honorary consultants)	-	-	4	14.29

### Physiotherapists’ knowledge of unilateral spatial neglect

The overall knowledge score on USN was 14.11 ± 5.23 (on a total scale of 25). Twenty-two (81.48%) physiotherapists had a moderate knowledge (score between 10 and 19), whilst only 2 (7.41%) had a good knowledge score (score of 20–25) on USN. Table 2 highlights the ranking of the score of physiotherapists’ knowledge of post-stroke USN.

**TABLE 2:** Ranking of scores on physiotherapists’ knowledge of post-stroke unilateral spatial neglect.

Variable	Value		
	Mean (SD)	*n*	%
**Overall knowledge about unilateral spatial neglect**	14.11 (5.23)	-	-
Maximum knowledge score	-	22	-
Minimum knowledge score	-	0	-
**Categories of USN knowledge score**			
< 10 Poor knowledge	-	3	11.11
10–19 Moderate knowledge	-	22	81.48
20–25 Good knowledge	-	2	7.41

SD, Standard deviation; USN, unilateral spatial neglect.

Analysis of the relationship between respondents’ demographics and USN knowledge showed that there was a significant low positive correlation between respondents’ age (*r* = 0.46; *p* = 0.016) and years of practice as a physiotherapist (*r* = 0.43; *p* = 0.026) and knowledge of USN. Conversely, respondent’s level (*p* = 0.066), working full time in neurorehabilitation (*p* = 0.918), possession of post-graduate certification in neurological rehabilitation (*p* = 0.126), clinical practice setting (*p* = 0.051), educational level (*p* = 0.651), gender (*p* = 0.595) and setting of facility (urban vs. semi urban) (*p* = 0.967) were not significantly associated with USN knowledge score of respondents. Table 3 shows the relationship between physiotherapists’ USN knowledge score and the demographics of respondents.

**TABLE 3:** Relationship between physiotherapists’ unilateral spatial neglect knowledge demographics.

Variable	Mean knowledge score	SD	Statistics	*p*
Age	14.11	5.23	0.46†	0.016**
**Gender**				
Male	15.67	3.79	0.54‡	0.595
Female	13.92	5.41	-	-
**Setting of the facility**				
Semi-urban	14.20	5.97	0.04‡	0.967
Urban	14.09	5.20	-	-
**Educational level**				
Bachelors	12.43	5.65	0.86§	0.651
Post-graduate diploma	0			
Master’s Degree	16.71	4.11	-	-
Doctorate	15.00	4.52	-	-
**Clinical practice setting**				
Hospital	10.00	5.79	7.77§	0.051
Rehabilitation centre	15.42	4.08	-	-
Out-patient Department	18.00	0.82	-	-
Domiciliary/Home health	17.00	2.83	-	-
**Postgraduate certification in neurological rehabilitation**				
Yes	16.50	4.38	−1.58‡	0.126
No	13.11	5.33	-	-
**Work full time in neurorehabilitation**				
Yes	15.80	3.82	−0.10‡	0.918
No	15.60	3.21	-	-
**Duration of practice in neurorehabilitation unit**	14.11	5.23	0.25†	0.377
**Years of practice as a physiotherapist**	14.11	5.23	0.43†	0.026¶
**Level**				
Physiotherapist	7.25	8.38	5.44§	0.066
Chief physiotherapist	16.20	2.77	-	-
Others (academics who are honorary consultants)	15.06	3.78	-	-

SD, standard deviation.

† , Spearman’s rank correlation;

‡ , *t-*test;

§ , ANOVA;

¶ , statistical significance.

Table 4 shows the item-by-item response to questions on USN neglect. The respondents demonstrated good knowledge in questions related to definitions of USN with 92.86% of respondents agreeing to question 1 (which is the correct response obtainable). Similarly, respondents demonstrated a moderate knowledge on questions about incidences and occurrence of USN (questions 2 and 7), causes of USN (question 5 and 6), anatomy of neglect (questions 3), prognosis (question 8, 9 and 10), screening and diagnosis (questions 11, 12, 13, and 14). However, respondents showed poor knowledge of the pharmacological approach to the management of USN (questions 15 and 16).

**TABLE 4:** Item-by-item frequency distribution of responses of physiotherapists to questions on knowledge on unilateral spatial neglect.

Number	Statement	Responses			
		Agree		Disagree	
		*n*	%	*n*	%
1.	Unilateral spatial neglect is the inability to orient or respond to stimuli appearing on the contralateral side of a brain lesion.	**26**	**92.86**	2	7.14
2.	Unilateral spatial neglect in stroke patients is more common in left hemispheric stroke than in right hemispheric stroke.	19	67.86	**9**	**32.14**
3.	Unilateral spatial neglect in stroke is commonly associated with a lesion in the inferior parietal lobe.	**13**	**48.15**	14	51.85
4.	Unilateral spatial neglect in stroke is commonly associated with cognitive dysfunction.	**15**	**55.56**	12	44.44
5.	Brain tumours can result in unilateral spatial neglect symptoms.	**23**	**85.19**	4	14.81
6.	Traumatic brain injury cannot result in unilateral spatial neglect symptoms.	23	85.19	**4**	**14.81**
7.	Unilateral spatial neglect in stroke is more common in younger patients than in older individuals.	16	59.26	**11**	**40.74**
8.	Most stroke patients with unilateral spatial neglect symptoms show recovery within the first week.	**1**	**3.70**	26	96.30
9.	Unilateral spatial neglect in stroke is associated with a longer hospital stay.	**19**	**70.37**	8	29.63
10.	Unilateral spatial neglect in stroke predicts poor rehabilitation outcomes.	**19**	**70.37**	8	29.63
11.	Albert’s test is a standardised screening tool for unilateral spatial neglect.	**12**	**44.44**	15	55.56
12.	The Crovitz-Zener scale can be used to screen for unilateral spatial neglect.	7	25.93	**20**	**74.07**
13.	Spinal cord injury is a condition to consider for differential diagnosis of spatial neglect.	23	85.19	**4**	**14.81**
14.	The best possible time for assessment of unilateral spatial neglect in stroke patients is at the chronic stage.	19	70.37	**8**	**29.63**
15.	Unilateral spatial neglect symptoms can be treated using pharmacological agents.	**2**	**7.41**	25	92.59
16.	The drug rivastigmine can be used in the management of unilateral spatial neglect symptoms.	**4**	**14.81**	23	85.19
17.	Mirror therapy is a rehabilitation option for unilateral spatial neglect.	**20**	**74.07**	7	25.93
18.	Eye patching is a rehabilitation option for unilateral spatial neglect.	**14**	**51.85**	13	48.15
19	Functional electrical stimulation and transcutaneous electrical stimulation are rehabilitation options for unilateral spatial neglect.	**14**	**51.85**	13	48.15
20.	Constraint-induced movement therapy is a rehabilitation option for unilateral spatial neglect.	**20**	**74.07**	7	25.93
21.	Line crossing, letter cancellation, star cancellation, figure and shape copying, line bisection and representational drawing can be used as an assessment tool to establish the presence of unilateral spatial neglect.	**24**	**88.89**	3	11.11
22.	Use of yoked prism is a treatment option for unilateral spatial neglect that its benefits extend to dressing, postural stability, walking, sit-to-stand transfers and wheelchair driving.	**13**	**48.15**	14	51.85
23.	Visual scanning exercise is not an effective technique in the treatment of unilateral spatial neglect.	13	48.15	**14**	**51.85**
24.	Listening to music scale will not ameliorate unilateral spatial neglect symptoms.	7	25.93	**20**	**74.07**
25.	Mental practice cannot improve unilateral spatial neglect symptoms.	16	59.26	**11**	**40.74**

Note: Bold responses depict the correct response for each question.

### Physiotherapists’ practice of post-stroke unilateral spatial neglect rehabilitation

Most of the respondents (*n* = 20; 71.43%) provide treatment to stroke survivors who exhibit spatial neglect symptoms. Nine (45%) respondents reported that they identified a maximum of two post-stroke USN patients every 3 months, whilst four respondents reported identifying about 16–20 post-stroke USN patients within the same time frame. Duration before initial evaluation to identify USN was also assessed. Nine respondents (45%) reported that it took about 1 to 2 days after stroke, on an average, before evaluating to identify USN. Similarly, 19 (95%) respondents reported performing a re-evaluation for USN after the initial evaluation to identify USN and 6 (31.58%) respondents reported that they performed re-evaluation about 6–10 days after they had performed an initial evaluation for USN (Table 5).

**TABLE 5:** Physiotherapists’ practice in post-stroke unilateral spatial neglect management.

Statement	Frequency	Percentage
**Do you provide treatment to stroke survivors who exhibit spatial neglect symptoms?**		
Yes	20	71.43
No	8	28.57
**Number of cases of stroke patients with unilateral spatial neglect you identify every 3-month period**		
1–2	9	37.50
> 2–5	9	37.50
> 5–10	1	4.16
> 10–15	1	4.16
> 15–20	4	16.66
**Duration before performing an initial evaluation to identify unilateral spatial neglect in stroke patients (days)**		
1–2	9	45.00
> 2–5	3	15.00
> 5–10	3	15.00
> 10–15	2	10.0
> 15–20	1	5.00
> 20–30	2	10.00
**Do you re-evaluate a patient for unilateral spatial neglect after you have performed an initial evaluation?**		
Yes	19	95.00
No	1	5.00
**How soon do you re-evaluate a patient for unilateral spatial neglect after you have performed an initial evaluation (in days)?**		
1–2	4	21.05
> 2–5	5	26.32
> 5–10	6	31.58
> 10–15	3	15.79
> 15–20	0	0
> 20–30	1	5.26
**Specific screening tool in asse ssing your patients for unilateral spatial neglect**		
Yes	10	50.00
No	10	50.00
**Do you refer patients with post-stroke to other members of the healthcare team?**		
Yes	20	100.00
No	0	0.00
**To whom do you refer?**		
Neurologist	7	25.00
Audiologist	6	21.43
Occupational therapist	19	67.86
Stroke specialist nurse	4	14.29
Speech therapist	13	46.43
Neuropsychologist	10	35.71
Cardiologist	7	25.00
Neuro-optometrist	4	14.29
Orthotist	1	3.59

**FIGURE 1:**
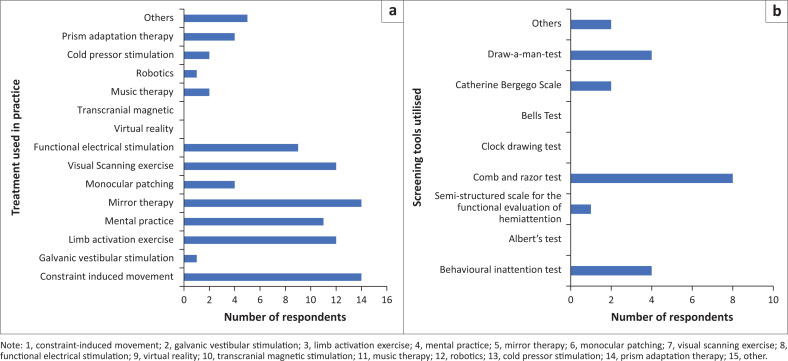
(a and b) Treatment and screening tools utilised by physiotherapists in practice. Physiotherapists’ perceived barriers and enablers to the treatment of post-stroke unilateral spatial neglect.

On screening for USN, half of the respondents reported using specific standard tools in screening for USN. The comb and razor test (*n* = 8; 28.57%) was reported as the most utilised assessment tool for USN screening amongst the respondents, whilst CIMT (*n* = 14; 50%) and mirror therapy (*n* = 14; 50%) were reported to be the two most utilised treatment options for USN amongst the respondents (Figure 1 shows the treatment and screening tool utilised by physiotherapists in the treatment of post-stroke USN in practice). All the respondents reported referring stroke survivors with USN to other members of the healthcare multidisciplinary team (MDT) of which occupational therapists (*n* = 19;67.86%) and speech therapists (*n* = 13; 46.43%) were MDT members with the most referrals from physiotherapists (see Table 5).

**FIGURE 2:**
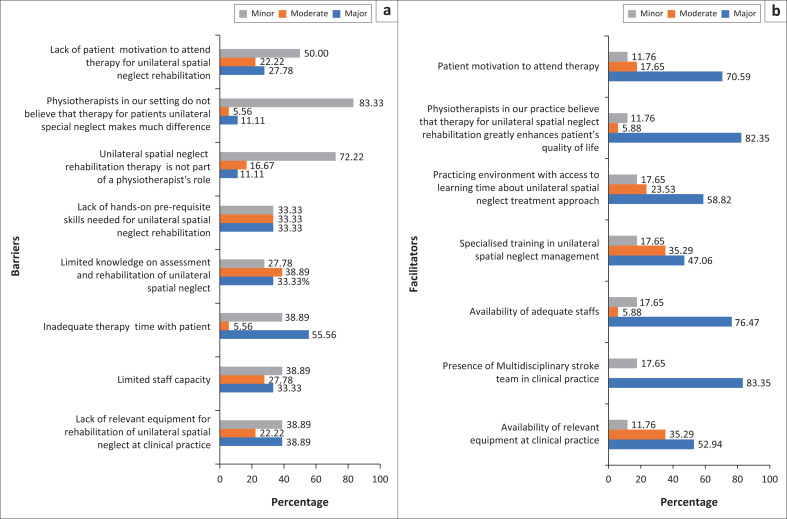
(a and b) Physiotherapist’s perceived barriers and facilitators to unilateral spatial neglect management.

When asked to identify barriers to USN rehabilitation, most of the physiotherapists (83.33%) reported that ‘physiotherapists in our setting do not believe that therapy for patients with USN makes much difference’ and was a minor barrier to USN rehabilitation. Also, another minor barrier to USN identification identified by a majority (72.22%) of the respondents was that ‘Unilateral spatial neglect rehabilitation therapy is not part of a physiotherapists role’. Conversely, ‘Inadequate therapy time’ (55.56%), ‘lack of relevant equipment for rehabilitation of unilateral spatial neglect in clinical practice’ (38.89%) were identified as major barriers to USN rehabilitation (Figure 2).

Major facilitators identified by the respondents included: ‘the presence of multidisciplinary stroke team in clinical practice’ (83.35%), ‘availability of adequate staff’ (76.47%) and ‘physiotherapists in our practice believe that therapy for USN rehabilitation greatly enhances patients’ quality of life’ (see Figure 2).

## Discussion

Physiotherapy is recognised as an autonomous profession in South Africa, accepting patients directly and as self-referrals (Bury & Stokes 2013). As such, physiotherapists practising in the South African health space are required to possess a sound theoretical, clinical and evidence-based knowledge of conditions they manage that includes post-stroke USN. Our study evaluated the knowledge on USN, current practice in the rehabilitation of USN and the barriers and enablers to USN rehabilitation amongst physiotherapists with a neurology bias in South Africa. Our study is the first to evaluate physiotherapists’ knowledge on USN, current practice in the rehabilitation of USN and the barriers and enablers to USN rehabilitation in South Africa.

The overall knowledge on the USN score of the respondents was moderate with only very few having poor knowledge of USN. Even though the general pooled knowledge score was adequate, item-by-item analysis of questions contained in the USN knowledge questionnaire showed some interesting results that might have some implications for training and practice. For instance, questions on pharmacological treatment for USN revealed that physiotherapists in South Africa had modest knowledge on the use of pharmacological agents in USN treatment. This may not be unrelated to the fact that physiotherapy training and legislation prohibit Physiotherapists to administer or prescribe medicines although many had a course in pharmacology during training. This is further corroborated by a study by Unger and Lochner (2006) that examined pharmacology practice amongst South African physiotherapists. In their study, respondents received theoretical training in pharmacology as part of their undergraduate qualification. This emphasises the need for a more in-depth integration of pharmacology in student training.

Respondents’ demographics and USN knowledge showed that there was a significant positive correlation between respondents’ age (*r* = 0.46; *p* = 0.016) and years of practice as a physiotherapist (*r* = 0.43; *p* = 0.026) and knowledge of USN. A previous study that investigated the knowledge of physiotherapists in South Africa on mental health (Hooblaul et al. 2020) found no association between knowledge of mental health and years of practice as a physiotherapist. However, a study by Taukobong et al. (2015) on knowledge of South African physiotherapists on health promotion identified age and work experience as associative factors to the knowledge of health promotion. Several reasons might explain this observation in our study, for instance, exposure to training facilities, access to learning facilities, location of practice (setting). This is evident in the higher knowledge level of a physiotherapist who practices in semi-urban areas as opposed to their counterparts in urban areas. The high load of patients encountered in urban areas may have constrained the therapist from utilising learning facilities, and this may have affected the therapist’s knowledge.

Also, respondents demonstrated a moderate knowledge on questions about incidences and occurrence of causes of USN and anatomy of neglect. Physiotherapists possess an in-depth understanding of gross anatomy necessary for safe and effective clinical practice. A study by Shead and colleagues in 2018 on anatomy education for South African undergraduate physiotherapy students reported that anatomy is taught in all eight schools that train physiotherapists in South Africa, and dissection, prosection, plastinated models, surface anatomy and e-learning were available across faculties (Shead et al. 2018). These learning environments might have positively influenced physiotherapists’ knowledge of the anatomy of neglect.

In South Africa, post-stroke rehabilitation typically commences immediately after the stroke survivor attains medical stability (Krakauer et al. 2012). Identification of post-stroke USN is reported as being modest as less than half of the therapists identified about two post-stroke patients with USN symptoms. This could be because of not having a routine assessment for the identification of USN or because of a low prevalence of USN in health facilities across South Africa. However, this is beyond the scope of our study and could only be substantiated in a future study. Interestingly, reported evaluation from time of admission for post-stroke USN is in line with clinical guidelines that recommend screening for visual perception and USN within 48 h of a patient re-gaining consciousness (National Stroke Foundation 2010; Robinson 2009). The association of USN with poor functional outcomes has been substantiated by previous authors (Jehkonen et al. 2000); it is, however, important to identify USN early in the rehabilitation process that can result in positive decision-making such as highlighting the importance for further perceptual assessment and commencing treatment interventions involving cognitive rehabilitation during hospitalisation (Bowen & Lincoln 2007).

We also showed that half of the physiotherapists reported using standardised outcome measures in the assessment/screening of post-stroke USN. This is in direct contrast with a Canadian study, where a paltry 13% of stroke survivors were assessed using a standardised outcome measure (Menon-Nair et al. 2006).

The use of standard outcome measures is a familiar discourse in the South African rehabilitation space. Inglis and colleagues in a cross-sectional survey of physiotherapists on awareness of outcome measures amongst physiotherapists in South Africa reported that 84% of responding therapists reported using standardised outcome measures frequently (Inglis, Faure & Frieg 2008). Our results are surprising given the previous report of high utilisation of standardised outcome measures by South African physiotherapists. This may be because of time constraints or reduced therapy time and lack of knowledge in the use of condition-specific outcome measures.

The specific test identified for screening for USN was the Comb and Razor test, Draw-a-Man test and the Behavioural Inattention test. The previous multidisciplinary international survey had identified the Line Cancellation test as the most popular test used in the screening for USN (Checketts et al. 2020). However, we did not investigate the factors that influence the selection of specific screening tools. It might be because of the ease of administration of the Comb and Razor test, the reduced time in administering and no cost implication in obtaining the test as the Comb and Razor test takes approximately 5 min to administer (McIntosh et al. 2000). Another probable explanation for the popularity of the Comb and Razor test amongst South African neurological physiotherapists could be an institutional preference. Anecdotally, most physiotherapy practice centres in South Africa have a battery of recommended outcome measures institutionalised in those centres although McGinnis et al. (2009) had in a previous study reported that patients’ medical diagnosis and history could inform the choice of assessment tool selection for balance assessment post-stroke.

Constraint-induced movement therapy, mirror therapy, visual scanning exercise and limb activation were the top four popular interventions for USN identified. This observation is similar to a previous study by Chen et al. (2018) that identified visual scanning, active limb activation, sustained attention training and prism adaptation therapy as the top four popular interventions for USN. Similarly, a systematic literature review by Luauté et al. (2006) recommended, based on available evidence, the use of visual scanning exercise, trunk rotation, mental imagery and muscle neck vibration. The CIMT was the top intervention of choice in our study. This is not surprising as CIMT is currently considered to be the most effective treatment regimen in physiotherapy for improving upper paretic limb rehabilitation outcomes (Langhorne, Bernhardt & Kwakkel 2011; Veerbeek et al. 2014). It has been reported that increased mobility of the affected limb, especially in the affected hemispace, could reduce the manifestations of visual-spatial neglect (Robertson & North 1993). The reasons for selecting these interventions were not explored here. However, physiotherapist preference, patient profile information and alternating to other effective treatment have been pinpointed to be reasons for the selection of a particular intervention.

Finally, we investigated the enablers and barriers faced by South African neuro-physiotherapist in the treatment of USN. Identified major barriers included inadequate therapy time and lack of relevant equipment for rehabilitation of USN in clinical practice. This is similar to reports by Checkett and colleagues that highlighted various barriers to USN rehabilitation as time and equipment shortages, sub-optimal MDT collaboration and lack of knowledge (Checketts et al. 2020). A major challenge in the South African context is that there are few public rehabilitation facilities leading to overcrowding and long waiting times and delays (Sokhela et al. 2013). The overload may ripple to the shortening of individualised therapy time. Treatment for USN is usually tailored to individual’s needs and needs ample time for a one-on-one session with the therapist. Also, the other major barrier identified is not alien to the African region; poor facilities and equipment have been an age-long challenge for the African health landscape (African Development Bank 2013). In contrast, perceived major enablers included: presence of a multidisciplinary stroke team, therapists’ belief that USN greatly enhances stroke patients’ quality of life (QoL) and availability of staff. These identified barriers and enablers are critical in understanding how to plan for better USN rehabilitation outcomes in the South African rehabilitation space.

## Limitations of study

The small sample size could affect the reliability of the survey results. Moreover, most of the respondents completed this survey questionnaire in urban areas. This limits the generalisation of the findings in semi-urban and rural settings.

A probable reason for the low response rate may be that physiotherapists may have missed the emails (as the emails may have been delivered to their spam because the questionnaire was sent in bulk to all SASP neurology interest group members). Another possible reason for the low response rate may be because physiotherapists as a result of their workload spend minimal time at their desks or in front of a computer or smart devices and missed emails containing the survey questionnaire.

## Conclusion

Physiotherapists surveyed demonstrated a fair knowledge of USN although knowledge about pharmacological management of USN appears to be modest. Current practice in post-stroke USN by South African neuro-physiotherapists is based on current evidence and practice guidelines.
